# Editorial: Biodiversity and ecosystem-level function of the rhizosphere

**DOI:** 10.3389/fpls.2023.1278662

**Published:** 2023-09-08

**Authors:** César Marín, C. Guillermo Bueno, Jianqing Wang, Vasilis Kokkoris

**Affiliations:** ^1^Centro de Investigación e Innovación para el Cambio Climático (CiiCC), Universidad Santo Tomás, Valdivia, Chile; ^2^Amsterdam Institute for Life and Environment, Section Ecology & Evolution, Vrije Universiteit Amsterdam, Amsterdam, Netherlands; ^3^Instituto Pirenaico de Ecología, Spanish National Research Council (CSIC), Jaca, Spain; ^4^Key Laboratory for Humid Subtropical Eco-geographical Processes of the Ministry of Education, Institute of Geography, Fujian Normal University, Fuzhou, China

**Keywords:** rhizosphere, soil ecology and microbiome, multifunctionality, mycorrhiza, ecosystem function, ecosystem services, biodiversity

How soil biodiversity affects ecosystem functioning has long been a central issue in ecology (Darwin, 1881). Models encompassing measurements of microbial processes and biodiversity exhibit superior explanatory capabilities for terrestrial carbon and nitrogen cycling compared to models that only include environmental variables (Graham et al., 2016). However, soil biodiversity and ecosystem functioning have rarely been jointly investigated (Guerra et al., 2020).

Soil and rhizospheric ecologists are only starting to reveal the correlational and causal relationships between macro-organismal/microbial biodiversity and multiple ecosystem functions and services, concept deemed ‘ecosystem multifunctionality’ (Hector and Bagchi, 2007). Fortunately, research on such multifunctionality has increased over the last few years (**Figure 1**). For example, Delgado-Baquerizo et al. (2020) found that the biodiversity of all soil taxa is positively related to soil ecosystem multifunctionality (11 functions including pathogen control, soil respiration and phosphorous mineralization, among others) at a global scale. Soil ecosystem multifunctionality was also directly related to soil carbon and indirectly related to climate and plant-related variables (Delgado-Baquerizo et al., 2020). These positive associations between soil-rhizospheric biodiversity and ecosystem functioning are not only derived from correlational studies. When experimentally simplifying the complexity of soil microcosms (by increasingly sieving soil into smaller particles), both Wagg et al. (2019) and Romero et al. (2023) have consistently observed a reduction in all (about 9-10) ecosystem functions and multifunctionality.

**Figure 1:**
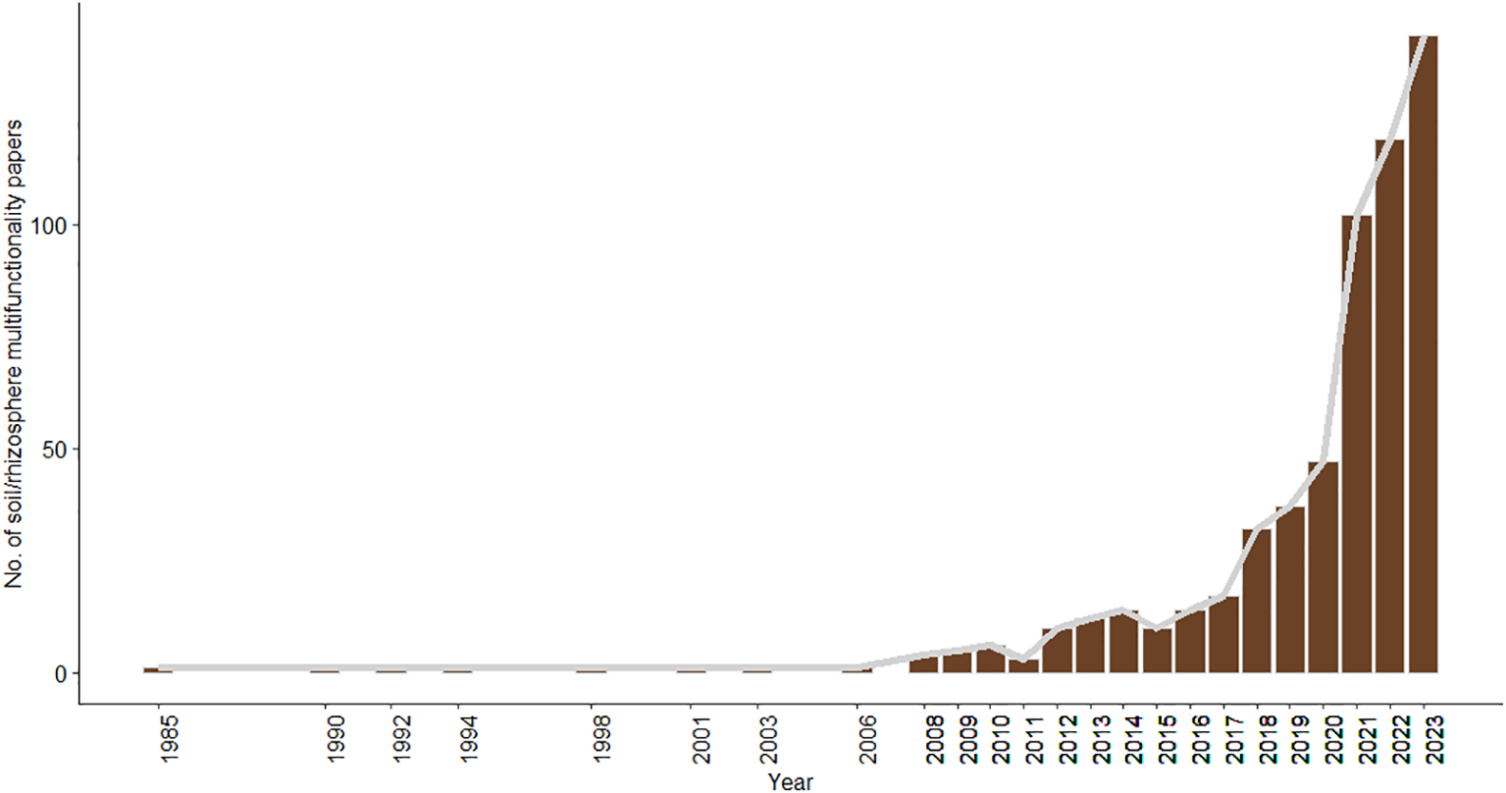
Number of SCOPUS-indexed English (research) articles on soil and rhizospheric ecosystem multifunctionality over the last years. Search terms in SCOPUS (7 August 2023): rhizosph OR soil AND multifunctionality.

Additional research paths on soil/rhizospheric biodiversity and ecosystem functioning relationships include contrasting global biogeographic patterns between flora and soil biota (Cameron et al., 2019; Vasar et al., 2022), understanding functional redundancy and why some ecosystem functions seem to be more related to certain taxa or guilds (Delgado-Baquerizo et al., 2020), and addressing methodological questions on how to accurately assess biodiversity and functions (Guerra et al., 2021). In this Research Topic, we addressed some of these research paths and other aspects related to biodiversity and ecosystem functioning of the rhizosphere. Wang et al. investigated the role of rhizospheric (locally and regionally) rare/abundant/entire bacterial and fungal communities on multiple ecosystem functions in monocultures and intercropped *Zea mays* croplands in subtropical China. At the local scale the authors found that rare bacteria were associated to ecosystem functions such as N-cycling multifunctionality and aboveground net primary productivity, whereas rare fungi were related to C and P cycling and overall ecosystem multifunctionality. Intercropping increased the network complexity and positive interactions of rare bacteria of the *Z. mays* rhizosphere. Also in an agronomic setting, Moura et al. found that neither different cover crops (in the Brazilian Cerrado ecosystem) nor N fertilization affected glomalin production and root colonization by arbuscular mycorrhizal fungi (AMF), and AMF spore density was slightly (yet significantly) affected by the different cover crop types evaluated. AMF diversity did not exhibit significant differences across cover crops. These results might be related to the ubiquity, low endemism levels (Davison et al., 2015; Davison et al., 2022), and high resilience of AMF communities, particularly those of the Cerrado (Moura et al., 2022).

Besides investigating abiotic and biotic drivers of AMF communities, Albracht et al. evaluated the effects of recurrent summer droughts (for 9 years) in experimental grasslands differing in plant richness and functional group richness. Plant functional composition, rather than mere plant species richness, shaped fungal communities during recurrent summer droughts. The aboveground components of grasslands also showed distinct patterns. As an illustration, Mao et al. discovered that six years of artificial afforestation led to the gradual development of grassland plant communities. These communities optimized composition, coverage, and aboveground biomass, with post-afforestation grassland diversity nearing that of a naturally recovering 10-year abandoned community.

As previously mentioned, understanding the global biogeography of soil biota remains a central concern in soil ecology. In line with this, Sepp et al. collected 327 soil samples around the globe. Their study employed metabarcoding sequencing of the *nifH* gene and the general prokaryotic 16S SSU region to delve into the global distribution of nitrogen-fixing bacteria in soils. Globally, the distribution of N-fixing bacteria was primarily influenced by climatic conditions, and soil pH and N content, with variations in these responses observed among specific taxa, such as Cyanobacteria. Similarly, using global-scale metabarcoding to elucidate global plant biodiversity, Vasar et al. found that large-scale plant diversity patterns and community composition elucidated through sequencing of 325 soil samples broadly coincided with the patterns revealed by traditional sources. Approximately half of local GBIF records were represented in this environmental DNA sequencing at the species level.

Changes in soil properties caused by plants, which in turn affect plant performance, termed ‘plant–soil feedback’ (PSF) (Van der Putten et al., 2013), are fundamental to understand relationships between soil biodiversity and functioning. Using a tree productivity gradient from long-term consistent reforestation experiments, Stefani et al. assessed the relationships between microbial communities and soil and tree nutrient stock, as affected by a positive PSF induced by wood mulch amendment in boreal forests. They found that adding such amendment resulted in a microbially-mediated positive PSF that increases mineral weathering and non-symbiotic N fixation. This shift turned unproductive plots into productive ones, contributing to rapid reforestation in a harsh environment. Similarly to Wang et al. regarding intercropping, Stefani et al. found that mulching increased the complexity and connectivity of soil microbial networks. Regarding the current effect of global warming, Wang and Shi calls out to the restoration-involved community, which encompasses forestry experts, legislators, and others, urging them to prioritize natural forest regeneration over different tree-planting techniques. They argue that such tree-planting techniques might have adverse impacts on soil and litter diversity, as well on certain crucial soil parameters such as organic matter, pH, nutrient/water retention, and overall structure.

In summary, the relationship between soil biodiversity and ecosystem functioning is gaining attention. Recent studies reveal correlations between biodiversity and multifunctionality, as well as the impact of factors like rare microbial communities and recurring droughts. These findings underscore the importance of PSF and pollution effects. This Research Topic contributes to understanding soil-rhizospheric interactions, highlighting their significance in ecosystem processes. As we advance, considering global biogeographic patterns, functional redundancies, and assessment methods will further enrich our comprehension.

## Author contributions

CM: Conceptualization, Formal Analysis, Writing – original draft, Writing – review & editing. CB: Conceptualization, Writing – review & editing. JW: Conceptualization, Writing – review & editing. VK: Conceptualization, Formal Analysis, Writing – review & editing.
